# A Heat-Killed *Cryptococcus* Mutant Strain Induces Host Protection against Multiple Invasive Mycoses in a Murine Vaccine Model

**DOI:** 10.1128/mBio.02145-19

**Published:** 2019-11-26

**Authors:** Yina Wang, Keyi Wang, Jorge A. Masso-Silva, Amariliz Rivera, Chaoyang Xue

**Affiliations:** a Public Health Research Institute, New Jersey Medical School, Rutgers University, Newark, New Jersey, USA; b Graduate School of Biomedical Sciences, New Jersey Medical School, Rutgers University, Newark, New Jersey, USA; c Department of Pediatrics and Center for Immunity and Inflammation, New Jersey Medical School, Rutgers University, Newark, New Jersey, USA; d Department of Microbiology, Biochemistry and Molecular Genetics, New Jersey Medical School, Rutgers University, Newark, New Jersey, USA; University of Massachusetts Medical School; Washington University School of Medicine

**Keywords:** *Cryptococcus neoformans*, Fbp1, fungal vaccine, invasive fungal infection, mice

## Abstract

Cryptococcus neoformans is a fungal pathogen that infects the lungs and then often disseminates to the central nervous system, causing meningitis. How *Cryptococcus* is able to suppress host immunity and escape the antifungal activity of macrophages remains incompletely understood. We reported that the F-box protein Fbp1, a subunit of the SCF(Fbp1) E3 ligase, promotes *Cryptococcus* virulence by regulating host-*Cryptococcus* interactions. Our recent studies demonstrated that the *fbp1*Δ mutant elicited superior protective Th1 host immunity in the lungs and that the enhanced immunogenicity of heat-killed *fbp1*Δ yeast cells can be harnessed to confer protection against a subsequent infection with the virulent parental strain. We therefore examined the use of heat-killed *fbp1*Δ cells in several vaccination strategies. Interestingly, the vaccine protection remains effective even in mice depleted of CD4^+^ T cells. This finding is particularly important in the context of HIV/AIDS-induced immune deficiency. Moreover, we observed that vaccinating mice with heat-killed *fbp1*Δ induces significant cross-protection against challenge with diverse invasive fungal pathogens, including C. neoformans, C. gattii, and Aspergillus fumigatus, as well as partial protection against Candida albicans. Thus, our data suggest that the heat-killed *fbp1Δ* strain has the potential to be a suitable vaccine candidate against cryptococcosis and other invasive fungal infections in both immunocompetent and immunocompromised populations.

## Abstract (precis)

Invasive fungal infections kill more than 1.5 million people each year, with limited treatment options. There is no vaccine available in clinical use to prevent and control fungal infections. Our recent studies showed that a mutant of the F-box protein Fbp1, a subunit of the SCF(Fbp1) E3 ligase in Cryptococcus neoformans, elicited superior protective Th1 host immunity. Here, we demonstrate that the heat-killed *fbp1*Δ cells (HK-fbp1) can be harnessed to confer protection against a challenge by the virulent parental strain, even in animals depleted of CD4^+^ T cells. This finding is particularly important in the context of HIV/AIDS-induced immune deficiency. Moreover, we observed that HK-fbp1 vaccination induces significant cross-protection against challenge with diverse invasive fungal pathogens. Thus, our data suggest that HK-fbp1 has the potential to be a broad-spectrum vaccine candidate against invasive fungal infections in both immunocompetent and immunocompromised populations.

## INTRODUCTION

Invasive fungal infections are emerging diseases that kill over 1.5 million people annually worldwide and are responsible for 50% of all AIDS-related deaths (1–3). As the immunocompromised population increases due to HIV infection, aging, and immunosuppressive treatments, including transplantation and chemotherapy, etc., the incidence of invasive fungal infections is expected to rise further. Because fungi are eukaryotes that share much of their cellular machinery with host cells, our armamentarium of antifungal drugs is highly limited, with only three classes of antifungal drugs available. Among them, polyenes are toxic, triazoles are fungistatic, and echinocandins have no effect against cryptococcal infections. With limited drug options and the emergence of drug resistance, there is an urgent need to develop new strategies to prevent and treat invasive fungal infections to ease the public health burden they cause. For many other infectious diseases caused by viruses and bacteria, vaccines have had a transformative impact on human health and wellbeing worldwide. There have been abundant studies focused on identifying fungal mutants and antigenic factors for potential fungal vaccine development (reviewed in references 4–6), and a vaccine against candidiasis has completed a phase II clinical trial (7, 8). However, despite heroic efforts, there are currently no vaccines in clinical use to combat fungal infections.

Cryptococcosis is the most common fungal disease in HIV-infected persons, and it can be caused by two species complexes: Cryptococcus neoformans and C. gattii. C. neoformans is a globally distributed pathogen that causes most cases of fungal meningitis in patients with HIV/AIDS, and it is responsible for more than 180,000 deaths annually (9–14). C. gattii is a primary pathogen that infects both immunocompromised and immunocompetent people. People with T cell immunodeficiency, such as HIV/AIDS patients, are highly susceptible to *Cryptococcus* infection, indicating the importance of cell-mediated immunity in host protection. Wild-type C. neoformans strain H99 expressing gamma interferon (IFN-γ; H99γ) has been shown to induce high Th1 immune response and provide full protection against virulent wild-type challenge, which also demonstrates the importance of cell-mediated immunity (15–17). Antibodies produced against glucuronoxylomannan (GXM) and other antigens have also been shown to be partially protective, indicating a protective role for humoral immunity (18, 19). Hence, both cell-mediated immunity and humoral immunity have been shown to be required for host defense against *Cryptococcus* infection.

In addition, C. neoformans mutant strains capable of inducing a highly protective Th1 response have been reported. Several mutant strains, such as a strain overexpressing transcription factor Znf2 (ZNF2^OE^), a chitosan-deficient *cda1Δ2Δ3Δ* strain, and a mutant lacking sterylglucosidase (*sgl1Δ*), have been identified as having increased immunogenicity in murine models. Their potential in vaccine development has also been proposed, and encouraging data have been reported (20–23). Besides the whole-cell-based vaccine strategy, simplified fungal antigenic factors, such as glucan particles, have also been identified for vaccine development (24, 25). All of these exciting developments suggest that a vaccine against *Cryptococcus* or other invasive fungal infections is feasible.

Early work from our laboratory identified an F-box protein Fbp1, a subunit of the SCF(Fbp1) E3 ligase, and characterized the importance of the SCF(Fbp1) E3 ligase-mediated ubiquitin-proteasome system in fungal development and virulence (26, 27). We recently showed that the *fbp1*Δ mutant can trigger superior Th1 protective immunity and found that both innate and adaptive immunity are involved in the host protection against *fbp1*Δ infection. CCR2 monocyte recruitment is essential for the *fbp1*Δ-mediated immune activation. A heat-killed *fbp1*Δ mutant (HK-fbp1) also induces high Th1 response, and mice vaccinated with HK-fbp1 cells show protection against the challenge of a virulent parental strain (28). These data indicate that the heat-killed *fbp1*Δ mutant has potential for development as a vaccine.

In this study, we further investigated the HK-fbp1-mediated host protection by optimizing the vaccine strategy and testing the specificity of vaccine protection. The data show that HK-fbp1 vaccination can trigger protection against multiple common invasive fungal infections, including C. neoformans, C. gattii, and Aspergillus fumigatus. It also showed partial cross-protection against Candida albicans in murine models. The protection against C. neoformans appears to work even in immunocompromised hosts, including animals lacking CD4^+^ T cells. We found that CD4^+^ T cell-depleted mice had increased CD8^+^ T cell recruitment and increased Th1 cytokine production to compensate for the loss of CD4^+^ T cells. We also found that IFN-γ signaling is required for HK-Fbp1-induced protection. Hence, our data indicate that the HK-fbp1 strain can be developed into a broad-spectrum fungal vaccine that provides protection against common invasive fungal infections in both immunocompetent and immunocompromised hosts.

## RESULTS

### The protection induced by HK-fbp1 vaccination against *Cryptococcus* is dose dependent.

**FIG 1:**
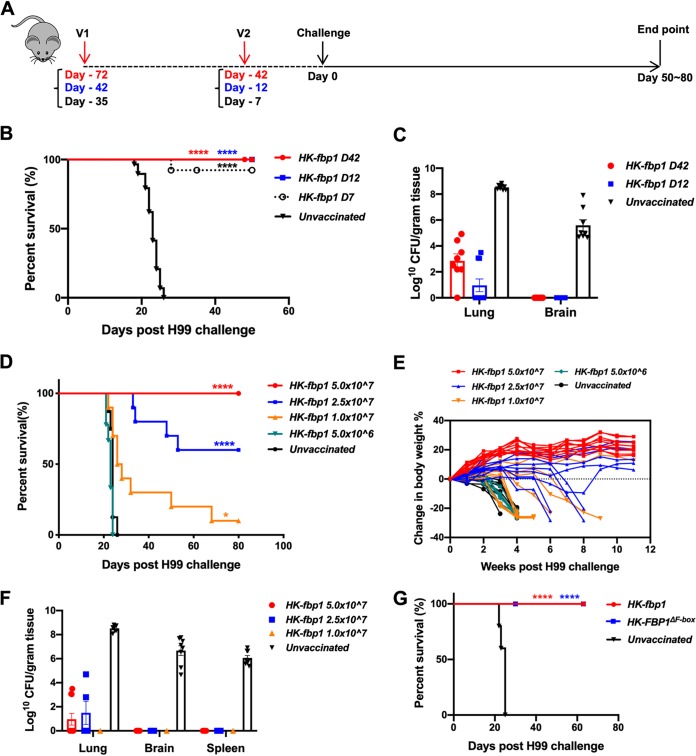
Protection induced by HK-fbp1 vaccination against *Cryptococcus* is dose dependent. (A) Scheme of vaccine strategy. V, vaccination. (B) Survival curves of mice vaccinated at different time points with the same dose of HK-fbp1 and challenged by infection with 10^4^ wild-type H99 cells. Eight to 10 female BALB/c mice were used for each group. ****, *P* < 0.0001. Statistical analysis of survival rates was determined by log rank (Mantel-Cox) test. (C) Fungal burden in the lungs and brains of vaccinated survivors at the end of the experiment. The endpoint fungal burden of unvaccinated mice was shown as a control. (D) Survival curves of mice vaccinated with different doses of HK-fbp1 and challenged with 10^4^ wild-type H99 cells. *, *P* < 0.05; ****, *P* < 0.0001 (determined by log rank [Mantel-Cox] test). (E) Changes in mouse body weight after challenge infection in each group. All living mice for each group were weighed and presented. (F) Fungal burden in the lungs, brains, and spleens of the surviving mice at the end of the experiment. The endpoint fungal burden of unvaccinated mice is shown as a control. (G) Survival curves of mice vaccinated with HK-fbp1 and heat-killed *fbp1Δ* strains expressing *FBP1* lacking the F-box domain (HK-Fbp1^ΔF-box^). ****, *P* < 0.0001 (log rank [Mantel-Cox] test).

Our recent study demonstrated that strong Th1 immune response developed in mice immunized with heat-killed *fbp1*Δ (HK-fbp1) cells. We also found that vaccination with HK-fbp1 induced protection against a subsequent challenge by a wild-type parental cryptococcal strain, while vaccination with the heat-killed wild-type strain did not (28). Here, we further examine the HK-fbp1-mediated vaccination strategy and the potential vaccine mechanism. In our previous study, vaccinated mice were challenged with wild-type parental strain H99 at 7 days postbooster vaccination (28). To optimize the time of challenge following vaccination and yield the best protection, we challenged mice at 7 days, 12 days, and 42 days postbooster vaccination (Fig. 1A). Our data show that while protection was established at all of these testing dates, challenges at 12 days and 42 days postbooster provide full protection (Fig. 1B). Therefore, we decided to use 12 days postbooster vaccination as the date of challenge infection in future studies.

We examined the fungal burden in the lungs and brains of the immunized mice at the endpoint of the challenge experiment. We did not detect any cryptococcal cells in the brains of vaccinated mice that were challenged by the wild-type H99 at 12 days and 42 days postbooster (Fig. 1C). The lungs of animals immunized with 5 × 10^7^ HK-fbp1 cells and challenged with 1 × 10^4^ H99 cells were either cleared of infection or contained significantly lower fungal burdens (Fig. 1C). This indicates that the protective immune response induced by vaccination is sufficient to clear or restrict H99 cells from dissemination during challenge infection. Taken together, our results demonstrate that immunization with the HK-fbp1 strain protects animals from subsequent lethal infection and restrains proliferation of wild-type H99 cells in the lungs.

We also examined the potential dose effect of the HK-fbp1 vaccine. Mice were vaccinated intranasally with different HK-fbp1 inocula (5 × 10^6^, 1 × 10^7^, 2.5 × 10^7^, 5 × 10^7^ cells/mouse) at day −42, given a booster vaccine at day −12 using the same dose, and challenged with H99 as described above. As expected, we found that the protection was dose dependent (Fig. 1D). Mice immunized with higher doses of vaccine exhibited better protection than those vaccinated with low doses (see Table S1 in the supplemental material). Mice vaccinated with 5 × 10^7^ HK-fbp1 cells conferred 100% protection, while no clear protection was observed for mice vaccinated with 5 × 10^6^ HK-fbp1 cells. We tracked the changes in animal body weight weekly throughout the experiment. As expected, we noticed that animals immunized with high doses of vaccine exhibited an increase in body weight over time, while those vaccinated with lower doses showed a gradual decline in body weight and eventually succumbed to the infection (Fig. 1E). These weight changes, which reflect overall animal health status, are another indication that mice were successfully vaccinated and well protected following vaccination with a high dose of HK-fbp1. Examination of the fungal burden in vaccinated mice that survived H99 infection showed brains and spleens were mostly cleared of fungal cells, while infected lungs had significantly lower fungal loads (Fig. 1F). Overall, our data suggest that a fungal antigenic threshold has to be reached in order for efficient vaccination with HK-fbp1 to induce lasting protection.

10.1128/mBio.02145-19.4 TABLE S1 Number of survival mice during the dose-dependent vaccination experiment Download Table S1, DOCX file, 0.01 MB. Copyright © 2019 Wang et al. 2019 Wang et al. This is an open-access article distributed under the terms of the Creative Commons Attribution 4.0 International license.

### Mice vaccinated with heat-killed Fbp1 lacking the F-box domain (HK-Fbp1^ΔF-box^) are protected from H99 challenge.

Fbp1 is a subunit of an SCF E3 ubiquitin ligase complex that is essential for fungal virulence (26, 27). Fbp1 binds to the other subunit, Skp1, via the F-box domain to form the SCF(Fbp1) protein complex, making the F-box domain essential for its E3 ligase function. To investigate whether the E3 ligase function is important for HK-fbp1-mediated immune activation, we generated a mutant strain that expresses the *FBP1* gene lacking the F-box domain (Fbp1^ΔF-Box^). Heat-killed cells of this mutant strain (HK-Fbp1^ΔF-box^) were prepared and used to vaccinate mice by following the same protocol as that described for Fig. 1A. Remarkably, vaccination with either HK-*fbp1*Δ or HK-Fbp1^ΔF-box^ conferred full protection, in contrast to the 100% mortality observed for nonvaccinated mice (Fig. 1G). This demonstrates that deletion of the F-box domain (thereby dissociating the SCF E3 ligase complex and eliminating E3 ligase function) is sufficient to mimic the *fbp1*Δ null mutant in stimulating protective immunity and providing vaccine protection against challenge with the virulent parental strain. These results also indicate that the E3 ligase function of Fbp1 is critical for modulating fungal antigenic factors important for host immune activation.

### Animals with depleted CD4^+^ T cells are protected by HK-fbp1 vaccination.

**FIG 2:**
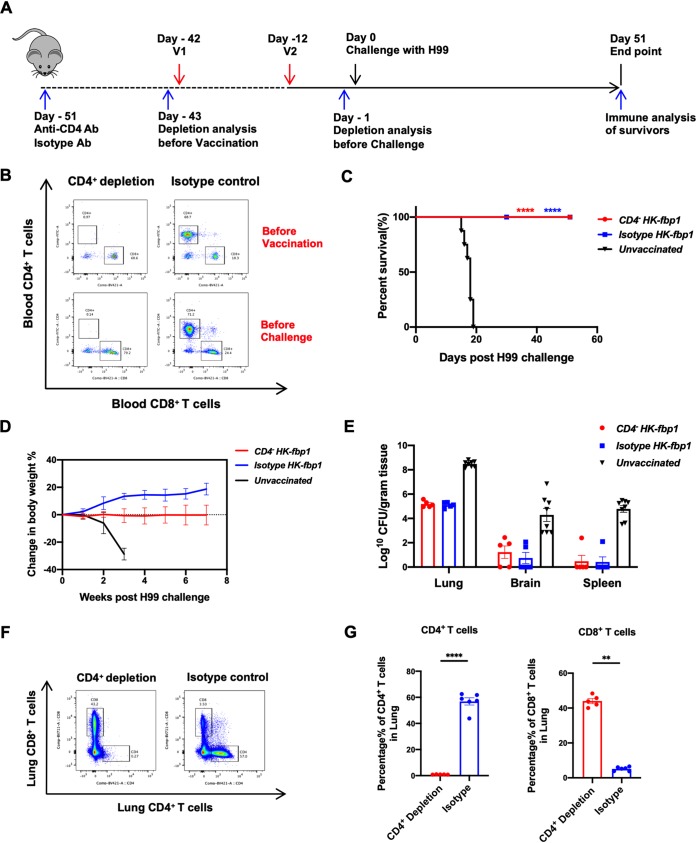
Animals with depleted CD4^+^ T cells remain fully protected by HK-fbp1 vaccination. (A) Strategy of vaccination. Depletion of CD4^+^ T cells was accomplished by injecting anti-CD4 antibody (GK1.5, rat IgG2b) weekly, starting 9 days prior to the first vaccination and weekly thereafter during the course of the experiment. (B) Detection of CD4^+^ and CD8^+^ T cells by flow cytometry prior to first vaccination and prior to challenge infection in mice injected with either CD4 antibody or isotype antibody. Representative FACS plots of CD4^+^ and CD8^+^ T cells in blood samples of CD4^+^ T cells-depleted mice and isotype control mice are shown. (C) Survival curves of CB/AJ mice vaccinated with HK-fbp1 and challenged by 5 × 10^4^ H99 cells. ****, *P* < 0.0001 (determined by log rank [Mantel-Cox] test). (D) Dynamics of body weight change in vaccinated and unvaccinated animals after challenge with 5 × 10^4^ H99 cells. All living mice from each group were weighted, and their average weight changes are presented. (E) Fungal burden in the lungs, brains, and spleens of vaccinated animals infected with 5 × 10^4^ H99 cells at the endpoint. The endpoint fungal burden of unvaccinated mice was shown as a control. (F) Efficient depletion of CD4^+^ and CD8^+^ T cells in mice was confirmed by using flow cytometry at the endpoint. Representative FACS plots of CD4^+^ and CD8^+^ T cells from lung tissues of CD4^+^ T cell depletion mice and isotype control mice are shown. (G) Cellular infiltration of the lungs was analyzed by flow cytometry at the endpoint. Each cell population was identified as CD45^+^, 4′,6-diamidino-2-phenylindole (DAPI)-negative live leukocytes. CD4^+^ T cells were gated as CD11b^−^ CD4^+^ CD8^−^, and CD8^+^ T cells were gated as CD11b^−^ CD4^−^ CD8^+^. ****, *P* < 0.0001; **, *P* < 0.01 (determined by Mann-Whitney test).

Individuals with immunodeficiency, such as impaired T cell function in HIV/AIDS patients, are highly susceptible to C. neoformans infection (9), and the importance of CD4^+^ T cells in defense against cryptococcosis has been well documented (29). Our previous study showed the involvement of CD4^+^ T and CD8^+^ T cells during induction of protective immunity by HK-fbp1 (28). Given the clinical significance of CD4^+^ T cell deficiency to the susceptibility to cryptococcosis in patients, it is critical to know whether host protection can be established following HK-fbp1 vaccination in CD4^+^ T cell-deficient hosts. To this end, we examined the potential vaccine protection in CD4-depleted mice. Depletion of CD4^+^ T cells was achieved by administration of 200 μg/mouse anti-CD4 antibody or isotype control antibody 9 days prior to first vaccination and weekly thereafter during the course of the experiment (Fig. 2A). Efficient depletion was confirmed by measuring the prevalence of CD4^+^ T cells in blood samples by flow cytometry on the day before the first vaccination (day −43) and the day before challenge (day −1) (Fig. 2B). Remarkably, CD4^+^ T cell-depleted mice remained highly protected by HK-fbp1 vaccination and were capable of mounting a protective response against challenge with H99 at two different inoculum levels, 1 × 10^4^ cells/mouse (Fig. S1) and 5 × 10^4^ cells/mouse (Fig. 2C). We tracked the changes in animal body weight weekly throughout the experiment. We noticed that both CD4^+^ T cell-depleted and isotype control animals challenged with H99 cells maintained or increased in body weight over time, while the unvaccinated ones lost weight rapidly following infection (Fig. 2D and Fig. S1). These weight changes indicated that CD4^+^ T cell-depleted mice were successfully vaccinated and protected against H99 challenge.

10.1128/mBio.02145-19.1 FIG S1 Animals with depleted CD4^+^ T cells remain fully protected by HK-fbp1 vaccination. (A) Survival curves of CBA/J mice vaccinated by HK-fbp1 and challenged with 1 × 10^4^ H99 cells. **** , *P* < 0.0001 (determined by log rank [Mantel-Cox] test). (B) Dynamics of average body weight changes to vaccinated and unvaccinated animals after challenge with 1 × 10^4^ H99 cells. All live mice from each group were weighed, and their average weight changes are presented. (C) Fungal burden in the lungs, brains, and spleens of vaccinated animals challenged with 1 × 10^4^ H99 cells at the endpoint of the experiment. The endpoint fungal burden of unvaccinated mice is shown as a control. Download FIG S1, TIF file, 0.6 MB. Copyright © 2019 Wang et al. 2019 Wang et al. This is an open-access article distributed under the terms of the Creative Commons Attribution 4.0 International license.

We further examined the fungal burden in lungs, brains, and spleens of the mice protected by immunization at the endpoint of the experiment. For animals challenged with 1 × 10^4^ H99 cells/mouse, no CFU were detected in most of the infected brains and spleens, while significantly lower numbers of CFU were recovered at 51 days postchallenge from infected lungs compared to the lungs of nonvaccinated animals (Fig. S1). For animals challenged with 5 × 10^4^ H99 cells/mouse, we again detected significantly reduced fungal burdens in the lungs of vaccinated animals. H99 cells were cleared from more than half of the brains and spleens of immunized animals during the period of this experiment, suggesting that immunization helped CD4^+^ T cell-deficient animals clear or restrict cryptococcal proliferation and dissemination in the lungs (Fig. 2E). The depletion was also confirmed by measuring the percentage of CD4^+^ T cells and CD8^+^ T cells in lung tissues by flow cytometry at the endpoint of the experiment (day 51) (Fig. 2F and G), while there was no significant change in monocytes and neutrophil recruitment (Fig. S2). These results suggest that vaccination with HK-Fbp1 can induce CD4^+^ T cell-independent protection. This finding supports the putative efficacy of HK-Fbp1 as an anticryptococcal vaccine candidate that could be given to immunocompromised patients (i.e., HIV-infected individuals).

10.1128/mBio.02145-19.2 FIG S2 Profile of the lung immunity (BAL and tissue homogenate). Cellular infiltration of the lungs was analyzed by flow cytometry. Each cell population was identified as CD45^+^, DAPI-negative live leukocytes. (A) Monocytes were gated as CD11b^+^ Ly6C^+^ Ly6G^−^. (B) Neutrophils were gated as CD45^+^ CD11b^+^ Ly6C^+^ Ly6G^+^. (C) Alveolar macrophages were gated as CD45^+^ CD11c^+^ SiglecF^+^. (D) Eosinophils were gated as CD45^+^ CD11c^−^ SiglecF^+^. Significance was determined by Mann-Whitney test. Download FIG S2, TIF file, 0.3 MB. Copyright © 2019 Wang et al. 2019 Wang et al. This is an open-access article distributed under the terms of the Creative Commons Attribution 4.0 International license.

### Role of CD4^+^ and CD8^+^ T cells and enhanced Th1 and Th17 T cell responses during the induction of protective immunity by HK-fbp1.

**FIG 3:**
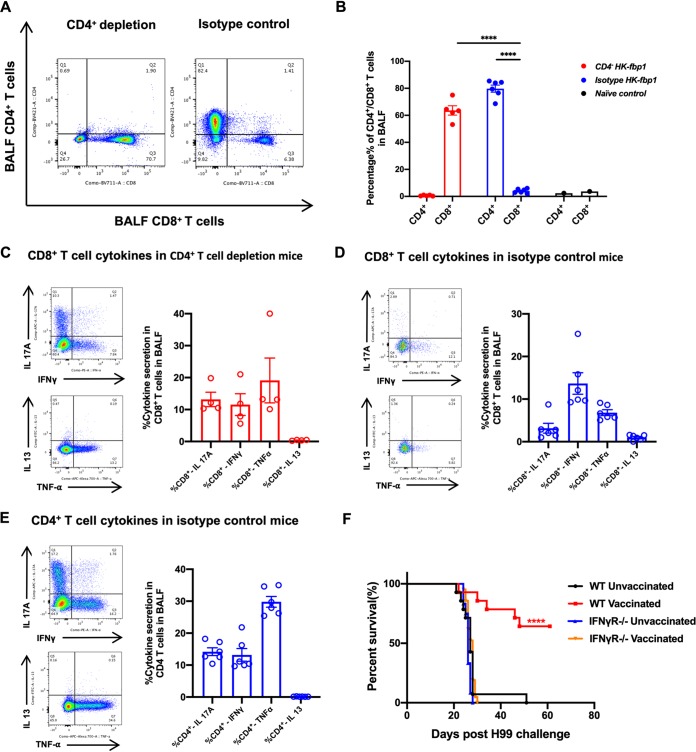
Role of CD4^+^ and CD8^+^ T cells and enhanced Th1 and Th17 T cell responses during the induction of protective immunity by HK-fbp1. (A) Representative FACS plots of CD4^+^ and CD8^+^ T cells from BALF of CD4^+^ T cell-depleted mice and isotype control mice at the endpoint of the experiment. (B) Percentage of CD4^+^ T cells and CD8^+^ T cells recovered from the BALF of survivors of CD4^+^ T cell-depleted and isotype control mice at the endpoint of the experiment. BALF cells were surface stained with fluorescently labeled antibody to Thy1.2, CD4, and CD8. CD4^+^ T cells gated as Thy1.2^+^ CD4^+^ CD8^−^ T cells. CD8^+^ T cells gated as Thy1.2^+^ CD4^−^ CD8^+^ T cells. Each symbol represents one mouse. Bars represent the means ± standard errors of the means. ****, *P* < 0.0001 (determined by Mann-Whitney test). (C and D) Cytokine production in CD8^+^ T cells from CD4^+^ T cell-depleted and isotype control mice at the endpoint. Representative FACS plots of CD8^+^ T cells are shown. Cytokine expression was analyzed by ICCS. Each symbol represents one mouse. Plots of cytokine production in CD8^+^ T cells gated as Thy1.2^+^ CD4^−^ CD8^+^ are shown. The frequencies of IL-17A-, IFN-γ-, TNF-α-, and IL-13-producing CD8^+^ T cells in BALF were analyzed as shown. (E) Cytokine production in CD4^+^ T cells from BALF of isotype control mice at the endpoint. Plots of cytokine production in CD4^+^ T cells gated as Thy1.2^+^ CD4^+^ CD8^−^ T cells. The frequencies of IL-17A-, IFN-γ-, TNF-α-, and IL-13-producing CD4^+^ T cells in BALF were analyzed as shown. Each symbol represents one mouse. (F) Survival curves of wild-type C57BL/6 mice (WT) and IFN-γ receptor-deficient mice (IFN-γR^−/−^) vaccinated with 5 × 10^7^ HK-fbp1 cells and challenged with 10^4^ H99 cells/mouse. A group of unvaccinated WT and IFN-γR^−/−^ mice was also infected by H99 as controls. ****, *P* < 0.0001 (determined by log rank [Mantel-Cox] test).

To understand how protection is established in CD4^+^ T cell-depleted mice, we examined the responses of remaining CD8^+^ T cell populations in vaccinated and infected mice at the endpoint of the experiment. We found that while the control mice had a significant increase in the number of CD4^+^ T cells in the infected lungs, CD4^+^ T cells were not detectable in the lungs of CD4^+^ T cell-depleted mice, as expected. Instead, we observed a significant expansion of CD8^+^ T cells in CD4^+^ T cell-depleted mice (Fig. 3A and B), a finding consistent with previous studies (30). We then analyzed the production of cytokines by airway-infiltrating CD8^+^ T cells using intracellular cytokine staining. Our data show that CD8^+^ T cells produced IFN-γ, tumor necrosis factor alpha (TNF-α), and interleukin-17A (IL-17A), with no induction of IL-13 (Fig. 3C and D). In the isotype control-treated mice, these cytokines were produced primarily by CD4^+^ cells (Fig. 3E). These results suggest that CD8^+^ T cells can compensate for the lack of CD4^+^ T cells in CD4^+^ T cell-depleted mice and are able to produce cytokines, such as IFN-γ, that are likely involved in HK-fbp1 vaccine-induced protection of the host.

In order to directly test whether IFN-γ is a critical mediator of vaccine-induced protection in this model, we examined the response to HK-fbp1 vaccination in IFN-γ receptor-deficient mice compared to control mice. Interestingly, mice defective in IFN-γ responsiveness were not protected after HK-fbp1 vaccination and were as susceptible to infection as unvaccinated mice (Fig. 3F). These data support the idea that the mechanism of HK-fbp1 vaccine-induced protection is dependent on the ability of this strain to induce a strong IFN-γ response in the host. Altogether, our findings also suggest that in the absence of CD4^+^ T cells, protective IFN-γ can be provided by CD8^+^ T cells.

### Mice vaccinated with HK-fbp1 are protected from challenge by C. gattii with sustained T cell response.

**FIG 4:**
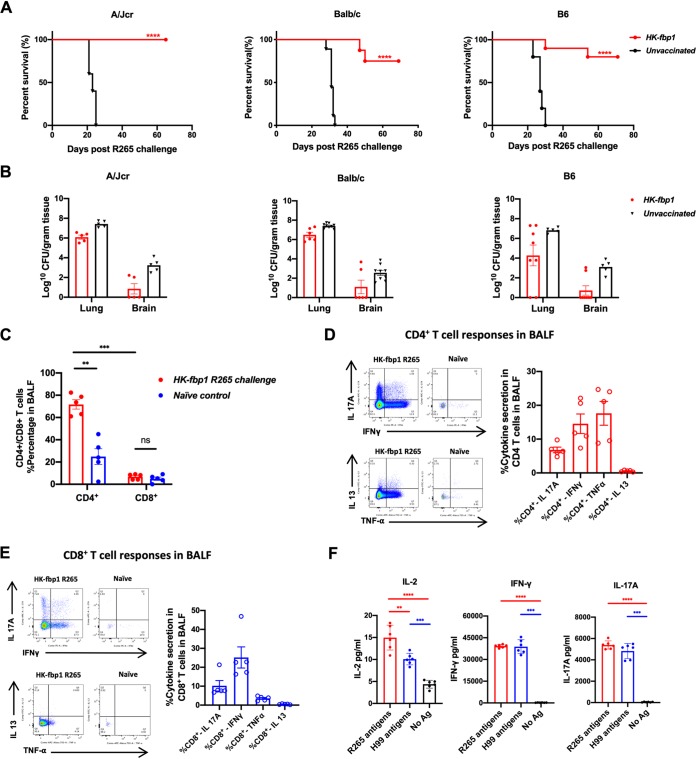
Mice vaccinated with HK-fbp1 are protected from challenge infection by *C. gatti* R265 and showed a sustained T cell response. (A) Survival curves of HK-fbp1-immunized A/Jcr, BALB/c, and C57BL6 mice challenged with C. gattii R265. ****, *P* < 0.0001 (determined by log rank [Mantel-Cox] test). (B) Fungal burden in the infected lungs and brains at the end of the experiment (∼65 days postchallenge). Each symbol represents one mouse. (C) Percentage of CD4^+^ and CD8^+^ T cells recovered from the BALF of survivors on day 65 after challenge with C. gattii R265. CD4^+^ T cells gated as Thy1.2^+^ CD4^+^ CD8^−^ T cells. CD8^+^ T cells gated as Thy1.2^+^ CD4^−^ CD8^+^ T cells. Each symbol represents one mouse. Bars represent the means ± standard errors of the means. ***, *P* < 0.001; **, *P* < 0.01 (determined by Mann-Whitney test). (D and E) Representative FACS plots of cytokine production of CD4^+^ T cells (D) and CD8^+^ T cells (E) in BALF of survivors. Cytokine expression was analyzed by ICCS. Plots of cytokine production in CD4^+^ T cells were gated as Thy1.2^+^ CD4^+^ CD8^−^ T cells. Plots of cytokine production in CD8^+^ T cells were gated as Thy1.2^+^ CD4^−^ CD8^+^ T cells. The frequencies of IL-17A-, IFN-γ-, TNF-α-, and IL-13-producing CD4^+^ T cells and CD8^+^ T cells in BALF were analyzed as shown. Each symbol represents one mouse. (F) *Cryptococcus*-specific CD4^+^ T cell responses were examined in lung-draining lymph nodes (MLNs) of HK-fbp1-vaccinated mice that survived an R265 challenge for 65 days. CD4^+^ T cells were purified and stimulated with antigen-presenting cells in the presence of C. gattii R265 or C. neoformans H99 antigens. The data shown are for cytokines secreted into the supernatant after 72 h of *in vitro* restimulation. Each symbol represents one mouse. The data were from an experiment with five or six mice per group and are depicted as the means ± standard errors of the means. ****, *P* < 0.0001; ***, *P* < 0.001; **, *P* < 0.01 (determined by one-way ANOVA).

Invasive cryptococcosis can be caused by infection with C. neoformans or C. gattii. In contrast to C. neoformans, C. gattii can infect immunocompetent populations and is considered a primary pathogen (31). Given the clinical importance of C. gattii, we tested the possibility of inducing cross-protection against C. gattii in mice vaccinated by HK-fbp1. Remarkably, vaccination with HK-fbp1 induced significant protection against challenge with infection by the highly virulent C. gattii clinical strain R265 in mice of three distinct backgrounds (Fig. 4A). A significant number of HK-fbp1-vaccinated mice survived for more than 60 days following challenge with 1 × 10^4^ R265 cells/mouse (Fig. 4A). Mice that survived for more than 60 days after infection were examined for fungal burden in the lungs and brains. While all mice contained low levels of R265 cells in the lungs, most of their brains were cleared of infection, with only a few containing low fungal CFU levels (Fig. 4B). At the endpoint of the experiment, mice that were protected by HK-fbp1 vaccination retained a significant percentage of CD4^+^ and CD8^+^ T cells in the airways (Fig. 4C) that produced IL-17A, IFN-γ, and TNF-α upon restimulation (Fig. 4D and E). The cytokine profile of T cells induced by HK-fbp1 vaccination was similar after challenge with H99 (Fig. 3) or R265, suggesting a conserved mechanism of protection. *Cryptococcus*-specific CD4^+^ T cell responses were examined in lung-draining lymph nodes (MLNs) of HK-fbp1-vaccinated mice that survived an R265 challenge for 65 days. CD4^+^ T cells were purified and stimulated with antigen-presenting cells in the presence of C. gattii R265 or C. neoformans H99 antigens. CD4^+^ T cells recovered from vaccinated mice rapidly produced IL-2, IFN-γ, and IL-17A upon restimulation with either H99 or R265, suggesting the activation of T cells that recognize antigens shared by both fungal strains (Fig. 4F). Altogether, these data suggest that HK-fbp1 vaccination is able to induce potent cross-protection against C. gattii in mice of diverse genetic backgrounds.

### Mice vaccinated with HK-fbp1 showed partial protection against challenge with fungal pathogen Candida albicans.

**FIG 5:**
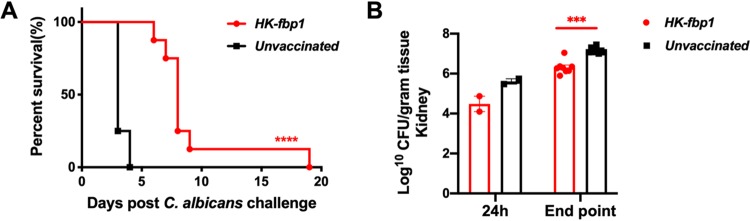
Mice vaccinated with HK-fbp1 showed partial protection against challenge by Candida albicans. (A) Survival curves of BALB/c mice vaccinated by HK-fbp1 and challenged with C. albicans strain CS3153. ****, *P* < 0.0001 (determined by log rank [Mantel-Cox] test). (B) Fungal burden in the kidneys of immunized and unimmunized animals at 24 h postinfection and at the end of the experiment. Each symbol represents one mouse. Bars represent the means ± standard errors of the means. ***, *P* < 0.001 (determined by Mann-Whitney test).

Candidiasis, caused by *Candida* species such as C. albicans, is another common invasive yeast infection. Our exciting observation of cross-protection offered by the HK-fbp1 vaccine against C. gattii prompted us to also test for potential cross-protection against C. albicans. Interestingly, although all vaccinated mice succumbed to *Candida* infection, a delay in death caused by C. albicans strain SC5314 was observed (Fig. 5A). While all unvaccinated mice developed terminal infection within 5 days, there was an average 5-day delay in the killing of vaccinated mice, indicating that the vaccination offered partial protection. Detection of fungal burden in infected mouse kidneys also showed an overall reduction in numbers of CFU at the endpoint of the experiment (Fig. 5B).

A *Candida* vaccine candidate (rAls3p-N) for treatment of recurrent vulvovaginal candidiasis has been reported to also protect vaccinated mice from *Staphylococcus* infection (32). Hence, we also tested the ability of our vaccine to protect mice from methicillin-resistant Staphylococcus aureus (MRSA) infection. Vaccinated mice were treated with cyclophosphamide 4 days prior to challenge with the MRSA strain to induce and maintain neutropenia, as described in Materials and Methods. Neither mouse survival rate nor bacterial numbers in CFU in infected kidneys, spleen, and liver showed significant protection following vaccination (Fig. S3).

10.1128/mBio.02145-19.3 FIG S3 Mice vaccinated with HK-fbp1 showed no clear protection against challenge by Staphylococcus aureus. (A) Survival curves of neutropenic mice vaccinated by HK-fbp1 and challenged with Staphylococcus aureus strain USA400. Mouse neutropenia was induced by cyclophosphamide injection. *, *P* < 0.05 (determined by log rank [Mantel-Cox] test). (B and C) Fungal burden in the kidneys, spleens, and livers of immunized and unimmunized animals at 24 h postinfection and at the endpoint of the experiment. Each symbol represents one mouse. Bars represent the means ± standard errors of the means. *, *P* < 0.05 (determined by Mann-Whitney test). Download FIG S3, TIF file, 0.4 MB. Copyright © 2019 Wang et al. 2019 Wang et al. This is an open-access article distributed under the terms of the Creative Commons Attribution 4.0 International license.

### HK-fbp1 vaccination offers cross-protection against Aspergillus fumigatus.

The cross-protection we observed in *Cryptococcus* species and C. albicans following HK-fbp1 vaccination prompted us to test the potential for cross-protection of our vaccine strain against the clinically relevant fungal pathogen Aspergillus fumigatus. A. fumigatus is a filamentous fungus that can cause deadly invasive fungal infections in immunocompromised patients. Drug-induced neutropenia is a significant risk factor for the development of invasive aspergillosis. Therefore, we examined whether HK-fbp1 vaccination could induce some degree of protection from invasive aspergillosis in a model of drug-induced neutropenia.

**FIG 6:**
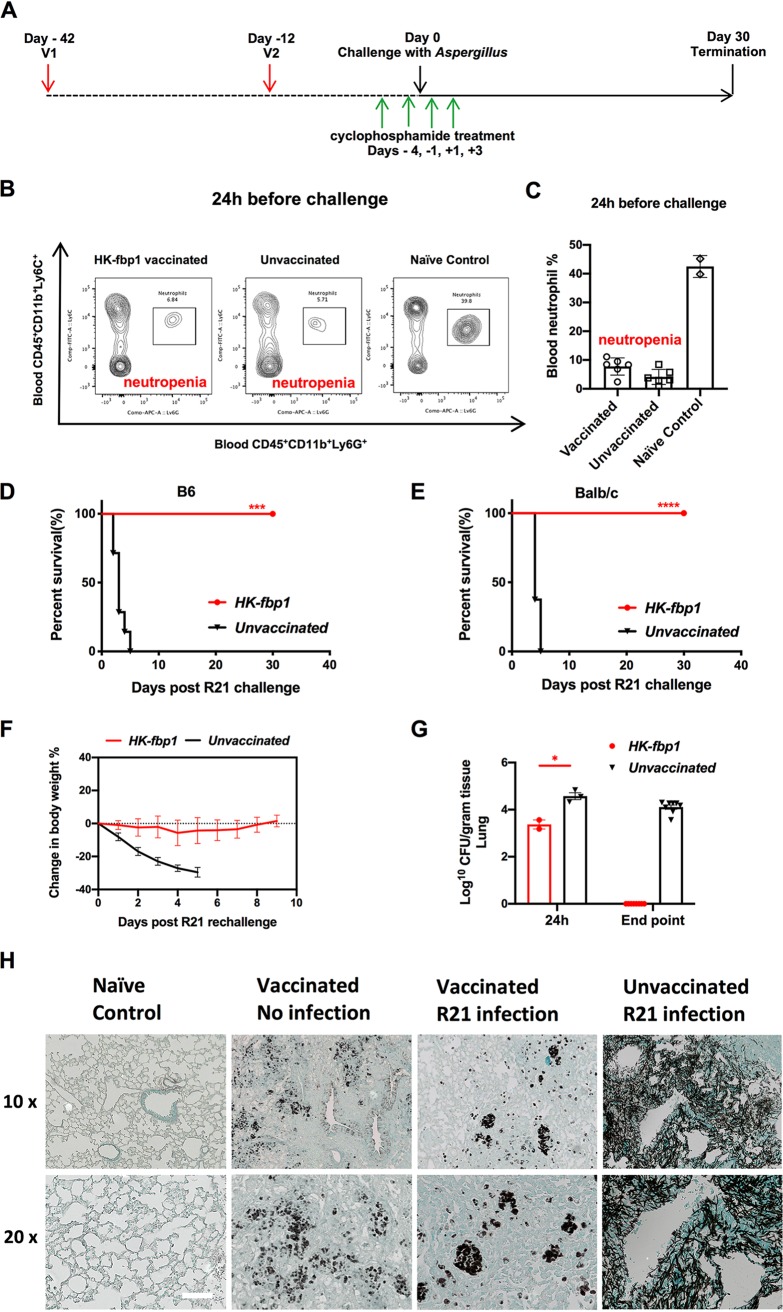
Heat-killed cells of *fbp1*Δ strain offered cross-protection against Aspergillus fumigatus in a murine model. (A) Strategy of vaccination and neutropenia induction. BALB/c mice were given an initial 150-mg/kg dose of cyclophosphamide via i.p. injection 4 days prior to infection (day −4). At day −1, day +1, and day +3, mice were given 100 mg/kg of cyclophosphamide i.p. to maintain neutropenia. (B) Representative FACS plots of neutrophils in blood samples of cyclophosphamide-treated HK-fbp1-vaccinated mice, unvaccinated mice, and control mice. Efficient neutropenia was confirmed by flow cytometric analysis 24 h prior to challenge with A. fumigatus. (C) Percentage of neutrophils from the blood samples of cyclophosphamide-treated HK-fbp1-vaccinated mice, unvaccinated mice, and control mice. Cellular infiltration to the blood samples was analyzed by flow cytometry. Each cell population was identified as CD45^+^, DAPI^−^ live leukocytes, and neutrophils were gated as CD11b^+^ Ly6C^+^ Ly6G^+^. Each symbol represents one mouse. (D and E) Survival curves of C57BL6 mice and BALB/c mice vaccinated by HK-fbp1 and infected with A. fumigatus strain R21. ***, *P* < 0.001; ****, *P* < 0.0001 (determined by log rank [Mantel-Cox] test). (F) Curves of mouse average body weight changes in each group. All live mice from each group were weighed, and their average weight changes are presented. (G) Fungal burden in the lungs of immunized and unimmunized animals at 24 h postinfection and at the endpoint of the experiment. Each symbol represents one mouse. Bars represent the means ± standard errors of the means. *, *P* < 0.05 (determined by Mann-Whitney test). (H) GMS-stained slides were prepared from infected lung tissue sections and visualized by light microscopy and photographed under ×10 and ×20 magnification. Bar indicates 20 μm.

Mice were vaccinated with HK-fbp1 by following our optimized regimen (Fig. 1). Four days prior to challenge, mice were treated with cyclophosphamide to deplete neutrophils and generate a state of drug-induced neutropenia in mice (Fig. 6A). The induction of neutropenia was confirmed by flow cytometric analysis using animal peripheral blood samples. Representative fluorescence-activated cell sorter (FACS) plots of neutrophil frequencies in the blood of cyclophosphamide-treated and untreated mice at 24 h before A. fumigatus strain R21 infection are shown (Fig. 6B). Each cell population was identified as CD45^+^, 4′,6-diamidino-2-phenylindole (DAPI)-negative live leukocytes, and neutrophils were gated as CD11b^+^ Ly6C^+^ Ly6G^+^ (Fig. 6B). We found that while the control mice had a normal level of neutrophils in the peripheral blood samples, neutrophils were maintained at low percentages in cyclophosphamide-treated vaccinated and unvaccinated mice, as expected (Fig. 6C). Interestingly, we observed protection in vaccinated neutropenic mice against challenge with the highly virulent A. fumigatus strain R21. In contrast, unvaccinated, neutropenic mice succumbed to A. fumigatus infection within a week. HK-fbp1-vaccinated mice of two distinct genetic backgrounds survived through to the endpoint of the experiment (more than 30 days) (Fig. 6D and E). Protected mice maintained a stable body weight during the experiment, while unvaccinated mice lost weight rapidly (Fig. 6F). Fungal burden in the lung was examined by CFU at 24 h in some of the experimental mice and at the endpoint of the experiment. We found that vaccinated mice completely cleared the *Aspergillus* infection, as no measurable CFU were recovered from the lungs of protected animals (Fig. 6G), in contrast to unvaccinated mice or vaccinated mice that were euthanized 24 h after A. fumigatus infection. Lung tissues were also examined by histology and stained with Grocott’s methenamine silver (GMS) to identify fungal structures in the pulmonary tissue of susceptible as well as protected mice. Unvaccinated mice had significant growth of *Aspergillus* hyphae in the lung (Fig. 6H), as expected. In contrast, HK-fbp1-vaccinated mice that survived the challenge with A. fumigatus did not contain hyphal structures in the lung but did retain stainable fungal material from the heat-killed yeast cells used for vaccination (Fig. 6H). We repeated this experiment three independent times with similar outcomes in mice of different genetic backgrounds (BALB/c and C57BL/6). Thus, our data indicate that HK-fbp1 vaccination can confer cross-protection against not only C. gattii but also A. fumigatus infection. This exciting observation suggests the activation of broadly protective antifungal mechanisms upon HK-fbp1 vaccination.

In summary, we have developed a murine vaccine strategy that employs HK-fbp1 as an effective vaccine strain against multiple invasive fungal infections in both immunocompetent and immunocompromised animals. These results support the utility of HK-fbp1 as a valuable research tool to understand mechanisms of fungus-host interaction and host antifungal immune activation. Our findings could have significant clinical implications that warrant further investigation and development.

## DISCUSSION

In this study, we examined the potential to apply the heat-killed *fbp1*Δ mutant strain (HK-fbp1) as a broad-spectrum fungal vaccine in murine models. We were encouraged by our earlier work that showed the significant immunogenicity of the *fbp1*Δ mutant (28). Our results demonstrate that vaccination with HK-fbp1 can provide protection against infection with C. neoformans even in the absence of CD4^+^ T cells. Moreover, HK-fbp1 vaccination can confer protection against infection with C. neoformans and its sibling species, C. gattii. Interestingly, we also observed full protection against challenge of Aspergillus fumigatus in vaccinated neutropenic mice and partial protection against C. albicans in wild-type mice. These findings are particularly important in the context of HIV/AIDS immune deficiency and suggest that the heat-killed *fbp1*Δ strain has the potential to be a suitable broad-spectrum vaccine candidate against cryptococcosis and other common invasive fungal infections.

Our previous work showed that vaccination with HK-fbp1 could induce significant host protection in both A/Jcr and C57BL/6 mice, yet the protection was not always 100% (28). Although there are various potential explanations for the variability in the efficacy of vaccine-induced protection, antigen dose and timing are potential factors that can be tested experimentally. Thus, in the current study we tested various vaccination regimens with diverse antigen doses and timing. Our findings suggest that prolonging the time between a booster dose and the infection challenge results in more efficient protection. The data further suggest that the protective immunity induced is long lasting and could be maintained in mice vaccinated 42 days prior to infection. As expected, we also found that the vaccination efficacy is dose dependent: administration of higher doses led to better protection. This dose dependency suggests that the immune-stimulatory factors that are differentially modulated in the *fbp1* mutant strain are low in abundance. Thus, increasing the number of killed yeast used in the vaccination helps improve the likelihood of proper immune stimulation. Studies aimed at identifying the specific targets that are modulated by the activity of Fbp1 will facilitate the future enrichment of protective factors. We anticipate that this ongoing work will allow for the future development of more simplified and effective HK-fbp1-derived vaccine components.

One potential limiting factor in fungal vaccine development is that invasive fungal infections occur more commonly in people with immunodeficiency, such as those with HIV/AIDS or neutropenic patients. Therefore, the goal in developing a fungal vaccine with maximum impact should be a vaccine that works in immunocompromised hosts. Multiple C. neoformans mutant strains have been reported to induce Th1 responses in mouse models of infection and to trigger host protection against challenge by virulent wild-type, parental strains (4, 15, 20–22). Among them, the IFN-γ-expressing H99 (H99γ) and a mutant strain lacking *SGL1* gene are avirulent in murine infection models and provide full protection against challenge by H99 in both wild-type and also CD4^+^ T cell-depleted mice (15, 22). However, in both cases, live cells are required to trigger immune protection. One potential risk of applying live attenuated strains to an immunocompromised human population is that weak host immunity may allow the vaccine strain to mutate and become infectious. Therefore, our observation that the HK-fbp1 cells conferred protection in CD4^+^ T cell-depleted mice and also in neutropenic mice is highly significant. This observation also begs the question: why are CD4^+^-depleted animals still protected? The detection of a significantly increased CD8^+^ T cell population in the CD4^+^ T cell-depleted mice following vaccination suggests that CD8^+^ T cells can compensate for the loss of CD4^+^ T cells. Our cytokine measurement confirmed this hypothesis. This is consistent with previous reports in other fungal infections (5) and suggests that a fungal vaccine is possible for immunocompromised populations, such as HIV/AIDS patients, who are highly susceptible to *Cryptococcus* infection.

Other heat-killed mutant strains have been reported to induce a high Th1 response, and mice vaccinated by these dead mutant cells were fully protected against C. neoformans challenge. Strains overexpressing ZNF2, a transcription factor involved in meiosis regulation, caused C. neoformans cells to be locked in the hyphal stage. Mice infected with heat-killed cells of this strain were also protected against infection of wild-type H99 (21). The *cda1*Δ*2*Δ*3*Δ triple mutant strain lacking the chitin deacetylase enzymes in C. neoformans blocked the production of chitosan, an important cell wall component, and both live and heat-killed *cda1*Δ*2*Δ*3*Δ mutant cells induced the Th1 response and triggered host protection against virulent wild-type infection (20). Whether these vaccine strains also can protect immunocompromised hosts remains to be determined.

Another important observation in our current study is that HK-fbp1 vaccination was able to induce significant protection against C. gattii infection. C. gattii is a primary pathogen that infects both immunocompromised and immunocompetent people. Cryptococcosis caused by C. gattii infection is more frequent in certain regions of the world and often is considered a regional disease. Therefore, the people in affected regions may benefit from the development of a broadly protective antifungal vaccine, such as HK-fbp1.

The strong protection observed against Aspergillus fumigatus is surprising, as it suggests the induction of cross-species antifungal protection. The exact mechanism of such cross-protection remains to be determined. Based on previous observations, there are several potential mechanisms for the induction of cross-protective immunity that could operate in a T cell-dependent or -independent manner. It is possible for fungi to contain common antigenic factors. In the case of cross-protection against C. gattii infection, it is possible, and indeed likely, for these two sibling strains to share many common antigens. Therefore, one can envision a scenario where vaccination with HK-fbp1 activates T cells with shared specificity against conserved *Cryptococcus* antigens. Previous work from the Klein laboratory nicely showed the existence of a T cell epitope commonly shared by *Ascomycetes* and the possibility for one T cell clone to protect against multiple fungal pathogens (33, 34).

Alternative mechanisms of vaccine-induced protection could operate independently of T cells. One such potential mechanism is based on the increased activation of innate cells by the HK-fbp1 vaccine, similar to what has been described as “trained immunity.” Distinct from well-studied adaptive immune memory, trained immunity has been described as a stimulus-dependent programming of innate cells of the myeloid lineage that are then able to respond more strongly to future stimuli with broad specificity (35, 36). Trained innate immunity-mediated vaccine protection was first proposed about a decade ago and has been confirmed in multiple vaccine studies, including the nonspecific protection of BCG vaccine (37–40). Recently, memory-like immune responses against C. neoformans challenge have also been reported in dendritic cells from mice vaccinated with H99γ, suggesting that innate immunity is trained to elicit long-lasting protective responses toward the resolution of infectious diseases (41). The induction of cross-protection after fungal infection has been one of the important bases for studies on trained immunity. Various studies have documented that the activation of dectin-1 by fungal cells can induce the epigenetic programming of monocytes and bone marrow precursors, inducing the long-term activation of broadly protective responses of innate cells (42–44). Intriguingly, our previous work showed that infection with *fbp1Δ* induces the increased recruitment and maturation of CCR2^+^ monocytes, and that these cells were required for protection from infection with *fbp1Δ* (28). Therefore, we hypothesize that upon vaccination with HK-fbp1, CCR2^+^ monocytes are trained to confer broad protection against other fungal pathogens. Future studies will be aimed at testing these exciting possibilities.

## MATERIALS AND METHODS

### Animals and fungal cultures.

Female mice with an average weight of 20 to 25 g were used throughout these studies. Mice of the A/Jcr, BALB/c, and C57BL/6 and its IFN-γ-R^−/−^ genetic backgrounds were purchased from the Jackson Laboratories, while mice of the CBA/J genetic background were purchased from Harlan Laboratories. Animal studies were performed at the Public Health Research Institute Animal facility. All studies were conducted by following biosafety level 2 (BSL-2) protocols and procedures approved by the Institutional Animal Care and Use Committee (IACUC) and Institutional Biosafety Committee of Rutgers University.

Cryptococcus neoformans clinical strain H99 and its mutants, as well as C. gattii strain R265 and Candida albicans SC5314, were cultured on yeast extract-peptone-dextrose (YPD) medium. Aspergillus fumigatus strain R21 was kindly provided by David Perlin and cultured on potato-dextrose agar (PDA) medium, and Staphylococcus aureus strain USA400 (MRSA) was cultured on LB medium.

### Vaccination strategy.

C. neoformans wild-type H99 and its mutant, *fbp1*Δ, were heat killed by following a previously described procedure (28). Fungal cells from YPD overnight cultures were precipitated and washed twice with sterile phosphate-buffered saline (PBS). The cell suspension with the correct concentration was then aliquoted into Eppendorf tubes and heated on a hot plate at 75°C for 90 min. The viability of the cells following heat treatment was examined by plating the processed cell suspension on YPD agar plates; no colonies were recovered after incubation at 30°C for 3 days. Mice were vaccinated intranasally with 5 × 10^7^ heat-killed fungal cells at day −42 unless otherwise specified. Each group of 8 to ∼10 mice was vaccinated again with the same dose of heat-killed fungal strains at day −12. A group of unvaccinated mice served as a control. The vaccinated groups and unvaccinated control group were challenged with either 1 × 10^4^ live H99 cells via intranasal inoculation or other fungal species as specified below. Infected animals were weighed and monitored daily for disease progression, and moribund mice were euthanized. All survivors were euthanized on day 65 after challenge with live H99 cells unless otherwise specified.

### Infection with cryptococci.

To prepare fungal cells for infection, overnight cultures of C. neoformans H99 or C. gattii R265 were washed three times with 1× PBS buffer, and the concentration of yeast cells was determined by hemocytometer counting. The final fungal concentration was adjusted with 1× PBS to 2 × 10^5^ cell/ml. For challenge infection, each mouse was infected intranasally with 1 × 10^4^ H99 or R265 cells in a 50-μl volume after being anesthetized with a mix of ketamine (12.5 mg/ml) and xylazine (1 mg/ml). After infection, animals were weighed daily and monitored twice daily for progression of disease, including weight loss, gait changes, labored breathing, and fur ruffling. Over the course of the experiments, animals that appeared moribund or in pain were euthanized by CO2 inhalation. Survival data from the murine experiments were statistically analyzed between paired groups by using the log rank (Mantel-Cox) test with PRISM version 8.0 (GraphPad Software, San Diego, CA) (*P* values of <0.05 were considered statistically significant). The change in body weight of each animal was calculated as [(weight on day X − weight on day 0)/weight on day 0] × 100%. The resulting data were plotted against time.

### Challenging with other fungal and bacterial species in murine models.

To test the efficacy of C. neoformans vaccination in protecting mice against other fungal and bacterial infection, 8-week-old BALB/c mice were vaccinated and challenged with Candida albicans, Aspergillus fumigatus, or Staphylococcus aureus (MRSA).

**(i) Candida albicans infection.** Vaccinated mice were infected with 1 × 10^5^ cells of C. albicans SC5314 via intravenous inoculation. This infection dose has been shown to be able to colonize and persist in the kidneys of the mice (28). The mice were closely monitored, being observed daily for any signs of illness. Any moribund mice were immediately humanely euthanized via CO_2_ narcosis, and fungal burdens were assessed. Kidneys were aseptically removed for enumeration of fungi to extrapolate the effect of vaccine.

**(ii) Aspergillus fumigatus infection.** Neutropenic mice (with severely reduced numbers of white blood cells) were used for A. fumigatus infection. To induce and maintain neutropenia in mice, vaccinated mice were given an initial 150-mg/kg dose of cyclophosphamide via intraperitoneal (i.p.) injection 4 days prior to infection. At day −1 prior to infection and day +1 and day +3 postinfection, mice were given 100 mg/kg of cyclophosphamide i.p. to maintain neutropenia. The mice were given sterile water and rodent chow containing doxycycline (DoxDiet) to prevent opportunistic bacterial infections and were housed in sterile cages throughout the study, including during the acclimation period. The immunocompromised mice were inoculated intranasally with a dose of 1 × 10^6^ spores in 25 μl of A. fumigatus R21 strain. Mice that developed a lethal infection were euthanized by CO_2_ inhalation, and lungs were harvested and assessed for microbial burden by quantitative CFU assay and histological assays.

**(iii) Staphylococcus aureus infection.** Neutropenic mice were used for the MRSA model (45) to evaluate the potential protection efficacy of HK-fbp1 vaccination. Mouse neutropenia condition was induced and maintained by cyclophosphamide injection as described above. Eight-week-old BALB/c mice were used for this study. The vaccinated mice were given 1 × 10^7^ cells of MRSA pathogen in 0.1 ml PBS via intravenous administration and were monitored for up to 5 days postinfection. The mice were weighed daily and closely observed for signs of morbidity, such as ruffled fur, labored breathing, inability to eat or drink, and mortality. Any mice displaying morbidity or weight loss of >15% were humanely euthanized. Microbial burden analyses of kidneys, spleen, and liver were performed to assess treatment efficacy of the vaccine. At the experimental endpoint, mice were euthanized by CO_2_ narcosis, and necropsies of major tissues (kidney, spleen, and liver) were performed.

### Histopathology and fungal burden in infected organs.

Infected animals were sacrificed at the endpoint of the experiment according to the Rutgers University IACUC approved animal protocol. To compare the fungal burdens and host inflammatory responses, the lungs, brains, and spleens were dissected and fixed in 10% formalin solution for section preparation at Rutgers University Histology Core Facility. Tissue slides were treated either with hematoxylin and eosin (H&E) staining for bronchus-associated lymphoid tissue or with Grocott’s methenamine silver (GMS) staining for fungal morphology observation *in vivo*. Infected lungs, brains, and spleens were also isolated and homogenized (Ultra-Turrax T8; IKA) in 3 ml cold 1× PBS buffer for 1 min for each type of organ. The tissue suspensions were serially diluted and plated onto YPD agar medium with ampicillin and chloramphenicol, and colonies were counted after 3 days of incubation at 30°C.

### CD4^+^ T cell depletion.

Mice were depleted of CD4^+^ T cell subsets via intraperitoneal administration of anti-CD4 (GK1.5, rat IgG2b) antibody (BE0003-1; BioxCell). Each mouse received 200 μg of GK1.5 or isotype (LTF-2, rat IgG2b) control (BE0090-A050MG; BioxCell) antibody in a volume of 200 μl PBS 9 days prior to the first vaccination and weekly thereafter during the observation period. Efficient depletion was confirmed by measuring the prevalence of CD4^+^ T cells in blood samples by flow cytometry on the day before the first vaccination (day −43) and the day before challenge (day −1). The depletion was also confirmed by measuring the prevalence of CD4^+^ T cells in bronchoalveolar lavage fluid (BALF) and lung tissues by flow cytometry at the endpoint of the experiment. The anti-CD4 antibodies used for flow cytometric analysis bind to the epitope of the CD4 protein at locations distinct from GK1.5. The RM4-4 fluorescein isothiocyanate (FITC) rat anti-mouse CD4 antibody (553055; BD Biosciences) was used for blood sample flow cytometric analysis, while the CD4 RM4-5 BV421 rat anti-mouse CD4 antibody (100543; BD Biosciences) was used for BALF and lung tissue flow cytometric analysis.

### Mouse genotyping.

Mouse genotyping was used to confirm the efficiency of CD4^+^ T cell depletion or the efficiency of neutropenia. Animal blood samples were processed for flow cytometry. Mice were lightly anesthetized by placing them in an isoflurane chamber. Blood samples from the end of mouse tails (2 to 3 drops/tail) were collected and placed into 50 μl of heparin (100 USP heparin units/ml) in a 96-well plate. After collection, bleeding was stopped by applying Kwik Stop styptic powder using a moistened cotton applicator to the end of the tail. Blood cells were washed with 150 μl of 1× PBS and resuspended in 200 μl of red blood cell (RBC) lysis buffer (155 mM NH_4_Cl and 10 mM NaHCO_3_), pH 7.2. Cells were washed again with 200 μl 1× PBS and resuspended in 50 μl of a 1:50 dilution of Fc block (CD16/CD32, 2.4G2) in FACS buffer (0.1% sodium azide in 1× PBS). After incubation on ice for 15 to 20 min, cells were washed with 150 μl of FACS buffer and resuspended in 50 μl of the appropriate antibody mix for each strain. Following 45 to 60 min of coincubation on ice, samples were washed with FACS buffer and resuspended in 200 μl of FACS buffer for flow cytometry. Cell surface antibodies CD45 (30-F11 allophycocyanin [APC]-Cy7), Thy1.2 (53-2.1 PE-Cy7), CD4 (RM4-4 FITC), and CD8α (53-6.7 Pacific Blue) were used to confirm CD4^+^ T cell depletion. Cell surface antibodies CD45 (30-F11 BUV395), CD11b (M1/70 peridinin chlorophyll protein [PerCP; Cy5.5), Ly6C (AL-21 FITC), and Ly6G (1A8 APC) were used to measure the prevalence of neutrophils in neutropenia mice.

### Lung processing.

Single-cell suspensions of pulmonary cells were prepared for flow cytometric analysis. In brief, lung tissue was minced in 5 ml of 1× PBS containing 3 mg/ml collagenase type IV (Worthington). Samples were incubated at 37°C for 45 min and washed with 1× PBS three times. After digestion, residual RBCs were removed using RBC lysis buffer (155 mM NH_4_Cl and 10 mM NaHCO_3_, PH 7.2). Total numbers of lung cells collected for each sample were determined by counting numbers of cells in 5 squares of a counting chamber using an inverted light microscope at ×40 magnification. Lung cell suspensions were used for flow cytometry. Lung single-cell suspensions were stained for monocytes (CD45 [30-F11 APC-Cy7], CD11b [M1/70 PerCP Cy5.5], and Ly6C [AL-21 PE]), Mo-DCs (CD45 [30-F11 APC-Cy7], CD11b [M1/70 PerCP Cy5.5], Ly6C [AL-21 PE], CD11c [N418 Pacific Blue], and MHC class II I-A/I-E [M5/11.415.2 Alexa Fluor 700]), neutrophils (CD45 [30-F11 APC-Cy7], CD11b [M1/70 PerCP Cy5.5], Ly6C [AL-21 PE], and Ly6G [1A8 APC]), CD4 T cells (CD45 [30-F11 APC-Cy7] and CD4 [RM4-5 Pacific Blue]), and CD8 T cells (CD45 [30-F11 APC-Cy7] and CD8α [53-6.7 FITC]). All antibodies used for lung staining were from BD Biosciences. All samples were analyzed using a BD LSRII or BD LSRFortessa flow cytometer and FlowJo software (Tree Star, Inc.).

### Intracellular cytokine staining of T cells harvested in BALF and flow cytometry.

For analyzing host immune responses, BALF samples were harvested at the endpoint after inoculation. BALF was collected in 3 ml of 1× PBS buffer using a catheter inserted into the trachea of animal posteuthanasia, and airway-infiltrating cells were lavaged with ∼0.7 ml of 1× PBS at a time to a total volume of 3 ml. RBCs were removed using RBC lysis buffer. BALF cells were then plated in a 96-well round-bottom plate and restimulated using BD-leukocyte activation cocktail containing BD GolgiPlug (BD Biosciences) according to the manufacturer’s instructions. Six hours after activation, BALF cells were surface stained with fluorescently labeled antibodies against Thy1.2, CD4, and CD8. Samples were fixed in 1% paraformaldehyde overnight. Prior to intracellular staining, the samples were permeabilized with 1× BD Perm/Wash buffer according to the manufacturer’s instructions. Intracellular cytokine staining (ICCS) was done using fluorescently labeled antibodies against IFN-γ, IL-17A, TNF-α, and IL-13 diluted in 1× BD Perm/Wash for 30 min on ice. Samples were immediately washed and analyzed by flow cytometry as described below. BALFs were cell surface stained for T cells with Thy1.2 (53-2.1 PE-Cy7), CD4 (RM4-5 BV421), CD8α (53-6.7 BV711) and ICCS for IFN-γ (XMG1.2 PE), IL-17A (eBio17B7 APC), TNF-α (MP6-XT22 Alexa Flour 700), and IL-13 (eBio13A FITC) by following standard procedures. Most antibodies and reagents for cell surface and ICCS were from BD Biosciences, except IL-17A and IL-13, which were obtained from eBioscience, Inc.

### CD4^+^ T cell isolation and CD4 T cell recall response.

Lung-draining lymph nodes (MLNs) were collected and placed in 10 ml of 1× PBS. Total lymphocyte cell suspensions were prepared by gently releasing the cells into the 1× PBS by applying pressure to the lymph nodes with the frosted ends of two glass slides. Repeated pressure was applied until the tissue was reduced to the smallest size possible. Samples were collected and processed in the same way individually. For CD4 T cell isolation, individual samples from each group were pooled (5 to 6 mice). CD4^+^ T cells were purified using a negative-sorting CD4^+^ isolation kit (Miltenyi Biotec, Inc., Auburn, CA). CD4^+^ T cell isolation was done by following the manufacturer’s instructions and were consistently found to be >90% pure, as assessed by flow cytometry. Purified CD4^+^ T cells (2 × 10^5^) were cultured with T cell-depleted antigen-presenting cells (3 × 10^5^) in RPMI containing 10% fetal calf serum (FCS), penicillin-streptomycin (2,200 U/ml; Gibco) and gentamicin sulfate solution (1 mg/ml). The cultures were plated in flat-bottom 96-well plates and incubated at 37°C with 5% CO_2_ for 72 h. Antigen-presenting cells were prepared from the spleen of syngeneic, uninfected donor mice. In brief, splenic cell suspensions were depleted of T cells by antibody complement-mediated lysis. Splenic cells were incubated with anti-Thy1.2 antibodies and rabbit complement (Low Tox; Cedarlane Labs, Hornby, ON, Canada) at 37°C for 60 min. To measure *Cryptococcus*-specific responses, CD4-antigen-presenting cell cultures were incubated with sonicated (Qsonica Sonicator Q55) H99 or R265 yeasts as a source of fungal antigens. The amount of antigen used was adjusted to a multiplicity of infection of 1:1.5 (antigen-presenting cell:yeast). The fungal growth inhibitor voriconazole was used at a final concentration of 0.5 mg/ml to prevent any fungal cell outgrowth during the culture period. After 72 h postculture, with initiation at 37°C with 5% CO_2_, supernatants were collected for cytokine analysis by enzyme-linked immunosorbent assay (IL-2 and IFN-γ, BD-OptEIA; IL-17A, Invitrogen) by following the manufacturers’ instructions.
