# Supplementary material for: A Heat-Killed Cryptococcus Mutant Strain Induces Host Protection against Multiple Invasive Mycoses in a Murine Vaccine Model
Source: mBio. 2019 Nov 26;10(6):e02145-19. doi: 10.1128/mBio.02145-19 (PMC6879717; doi:10.1128/mBio.02145-19)
Supplement: TABLE S1 [file mBio.02145-19-st001.docx]

Table S1 Dose-dependent vaccination experiment

| Vaccine dose |  | Number of animals | | |  |
| --- | --- | --- | --- | --- | --- |
|  | D0 | D20 | D40 | D60 | D80 |
| 5.0 x 10^7^ | 10 | 10 | 10 | 10 | 10 |
| 2.5 x 10^7^ | 10 | 10 | 8 | 6 | 6 |
| 1.0 x 10^7^ | 10 | 10 | 3 | 2 | 1 |
| 5.0 x 10^6^ | 9 | 9 | 0 |  |  |
| Unvaccinated | 8 | 8 | 0 |  |  |
