# Supplementary figures and images for: A Heat-Killed Cryptococcus Mutant Strain Induces Host Protection against Multiple Invasive Mycoses in a Murine Vaccine Model
Source: mBio. 2019 Nov 26;10(6):e02145-19. doi: 10.1128/mBio.02145-19 (PMC6879717; doi:10.1128/mBio.02145-19)

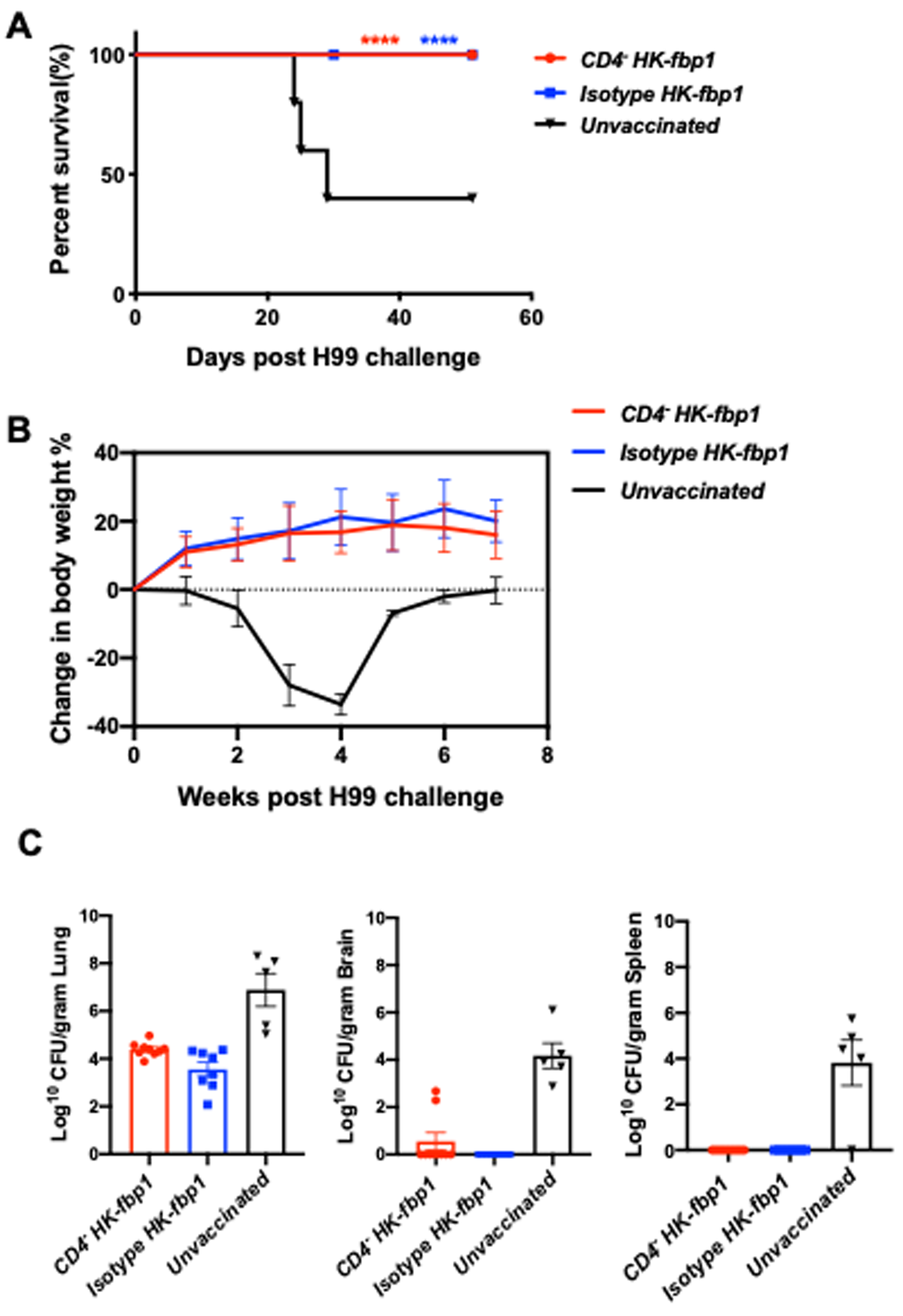

Supplement: FIG S1 [file mBio.02145-19-sf001.tif]

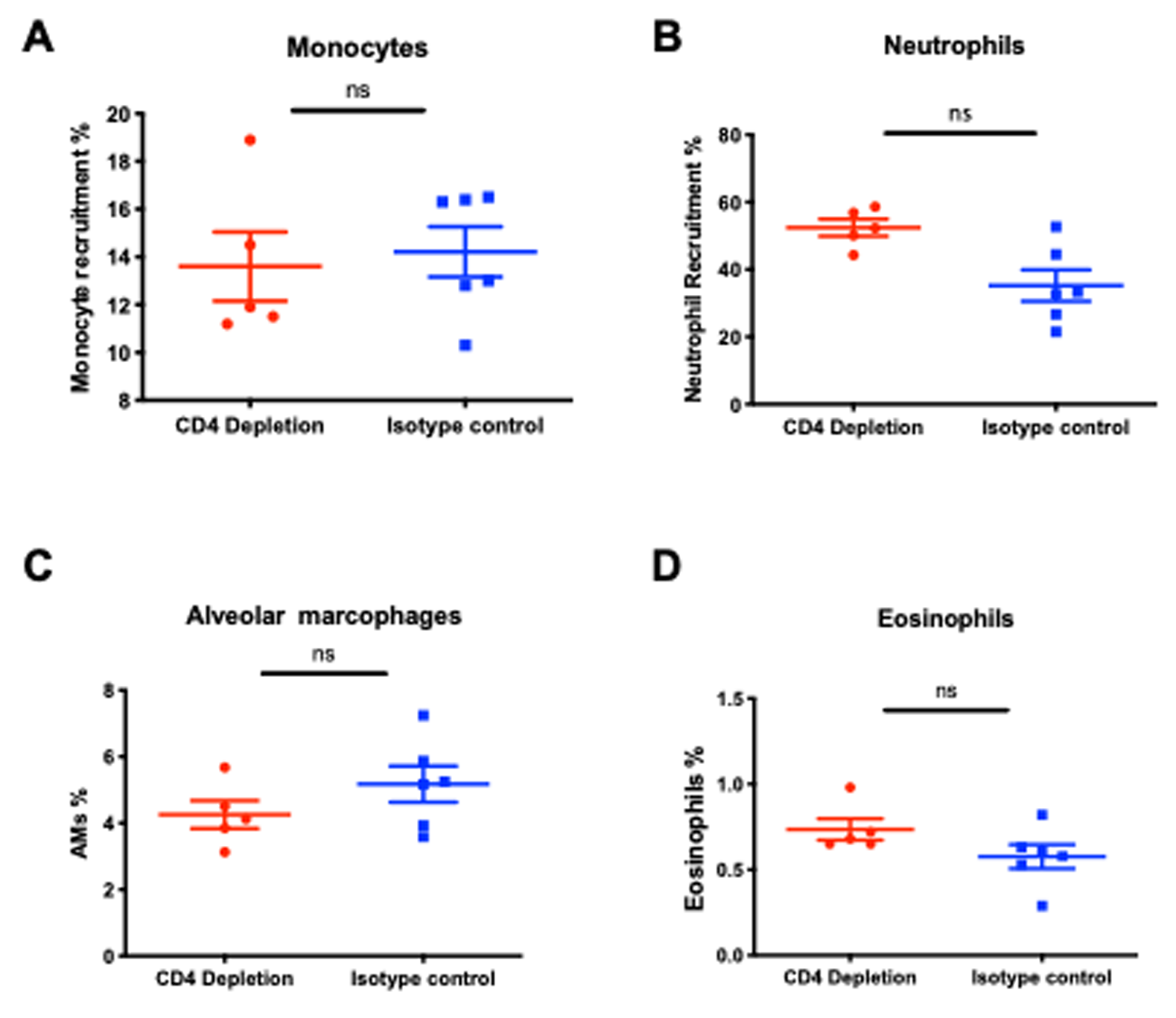

Supplement: FIG S2 [file mBio.02145-19-sf002.tif]

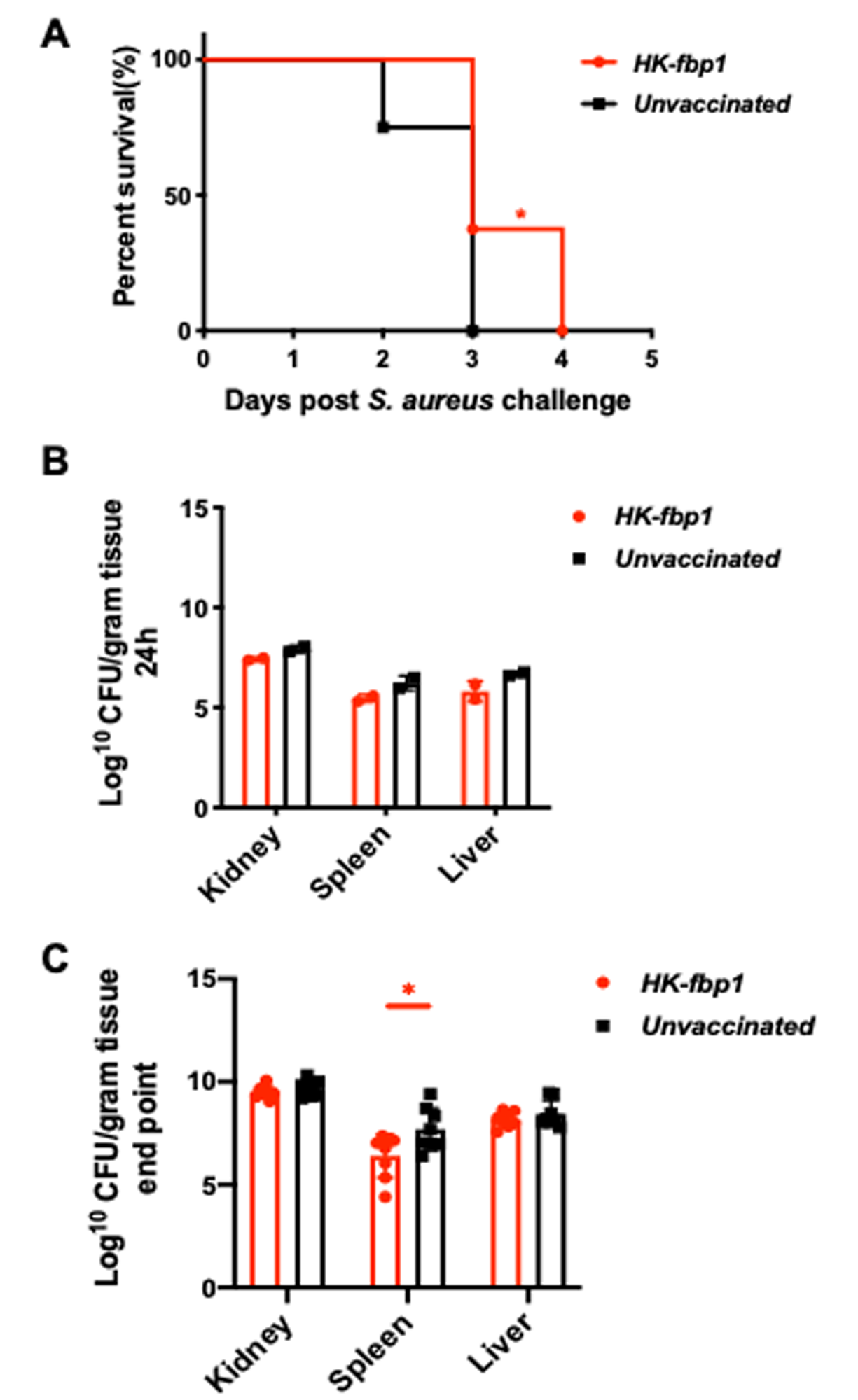

Supplement: FIG S3 [file mBio.02145-19-sf003.tif]
